# A novel splice site mutation in *WAS* gene in patient with Wiskott-Aldrich syndrome and chronic colitis: a case report

**DOI:** 10.1186/s12881-018-0647-0

**Published:** 2018-07-20

**Authors:** Hossein Esmaeilzadeh, Mohammad Reza Bordbar, Hassan Dastsooz, Mohammad Silawi, Mohammad Ali Farazi Fard, Ali Adib, Ali Kafashan, Zahra Tabatabaei, Forough Sadeghipour, Mohammad Ali Faghihi

**Affiliations:** 1 Allergy Research Center, Shiraz University of Medical Sciences, Shiraz, Iran; 2 Department of Allergy and Clinical Immunology, Namazi Hospital, Shiraz, Shiraz, Iran; 3 Hematology Research Center, Shiraz University of Medical Sciences, Shiraz, Iran; 4 Persian Bayan Gene Research and Training Institute, Faghihi Medical Genetics Center, Shiraz, Iran; 5 Italian Institute for Genomic Medicine (IIGM), University of Turin, Turin, Italy; 6 Department of Psychiatry and Behavioral Sciences, University of Miami Miller School of Medicine, Miami, USA

**Keywords:** Wiskott-Aldrich syndrome, Splice site mutation, *WAS* gene, Chronic colitis, Case report

## Abstract

**Background:**

Wiskott-Aldrich syndrome is an X-linked recessive immunodeficiency due to mutations in Wiskott-Aldrich syndrome (*WAS*) gene. *WAS* gene is encoded for a multifunctional protein with key roles in actin polymerization, signaling pathways, and cytoskeletal rearrangement. Therefore, the impaired protein or its absence cause phenotypic spectrum of the disease. Since identification of novel mutations in *WAS* gene can help uncover the exact pathogenesis of Wiskott-Aldrich syndrome, the purpose of this study was to investigate disease causing-mutation in an Iranian male infant suspicious of this disorder.

**Case presentation:**

The patient had persistent thrombocytopenia from birth, sepsis, and recurrent gastrointestinal bleeding suggestive of both Wiskott-Aldrich syndrome and chronic colitis in favor of inflammatory bowel disease (IBD). To find mutated gene in the proband, whole exome sequencing was performed for the patient and its data showed a novel, private, hemizygous splice site mutation in *WAS* gene (c.360 + 1G > C).

**Conclusions:**

Our study found a novel, splice-site mutation in *WAS* gene and help consider the genetic counselling more precisely for families with clinical phenotypes of both Wiskott-Aldrich syndrome and inflammatory bowel disease and may suggest linked pathways between these two diseases.

## Background

Wiskott-Aldrich syndrome (WAS), [MIM: 301000], which is a rare X-linked recessive immunodeficiency is mainly characterized by thrombocytopenia, eczema, infection, and bloody diarrhea. The disease usually leads to death before the age of 10 years [1–3]. Patients with WAS, usually, suffer from upper and lower respiratory tract infections, chronic diarrhea, melena and inflammatory bowel disease (IBD). The incidence of WAS throughout the world is estimated to be 1 to 10 in 1 million live birth per year [4]; however; its prevalence or incidence in Iran has not been reported yet. The disease is caused by pathogenic mutations in the *WAS* gene (located on Xp11.22–23) consisted of 12 exons. The gene is encoded for the Wiskott-Aldrich syndrome protein (WASp), a cytoplasmic 502–amino acid protein involved in the signal transduction from cell surface receptors to the actin cytoskeleton. Its main expression is in non-erythroid hematopoietic cells, indicating its essential roles in the function of these cells [5–7]. The WASp is a multifunctional protein with the key involvement in actin polymerization, signaling pathways, and cytoskeletal rearrangement, which is crucial for the monocytes and macrophages migration to infection sites and inflamed tissues, and binding and phagocytosis of antigens [1, 8, 9]. Thus, complete or partial deficiency of WASp leads to malfunctioning of tissue macrophages, neutropenia, and small platelet sizes, causing repeated infections as well as bleeding tendency [4].

Up to know, more than 431 mutations have been reported in *WAS* gene (Human Genome Mutation Database, HGMD, http://www.hgmd.cf.ac.uk). According to the HGMD database, most of WASp mutations have occurred within the 4 most N-terminal exons of the gene with the arginine residue at position 86 accounting for the most common mutated amino acid in WASP. Different mutations in this protein can cause variable severity of the disease. For instance, study conducted by Greer et al. [10] revealed that the WAS patients with milder clinical presentations had missense mutations. Moreover, study conducted by Villa et al. showed that some *WAS* mutations can cause only thrombocytopenia with small-sized platelets [2] but, up to now, it is not fully understood why specific disease-causing variants only affect the megakaryocytic cells. In addition, in a WAS family with thrombocytopenia, increased levels of serum IgA and mild nephropathy has been observed to complicate the pathogenesis of this disorder [11]. Since identification of different mutations in *WAS* gene may help understand the pathogenesis of WAS, the aim of this study was to identify pathogenic variation in our patient with clinical findings suspicious of WAS.

## Case presentation

An 8-month-old Iranian male infant, a product of consanguineous marriage, was admitted to our center with history of persistent thrombocytopenia from birth, sepsis, and recurrent gastrointestinal bleeding. In family history, the proband had a sibling who died with similar phenotypes. Initial laboratory findings at different ages were suggestive of idiopathic thrombocytopenic purpura (ITP) (Table 1), therefore; intravenous immunoglobulin (IVIG) was administered for him. At the age of 1 month, he showed mild skin thickening and bone marrow aspiration revealed moderate hypo-cellular marrow with decreased megakaryocyte. However, TORCH study, rheumatologic work up, and levels of complement components such as C3, C4, and CH50 were in normal range. At the age of 4 months, he had increased levels of erythrocyte sedimentation rate (ESR) and C-reactive protein (CRP) and decreased levels of hemoglobin (Hb) and mean platelet volume (MPV), indicating thrombocytopenia. Therefore, IVIG and platelet were administered for the patient.

**Table 1:** Results of complete blood count at different ages of the proband

Age	22 days	40 days	4 months	8 months	12 months
WBC (per mm3)	9200	17,300	3700	6600	12,100
RBC (per mm3)	3.36*10^6	_	3.35*10^6	2.82*10^6	4.36*10^6
Hb (gr/dl)	10.8	12	6.8	6.6	7.9
MCV (fl)	97	_	70.4	77	66.5
PLT (per mm3)	45*10^3	130*10^3	64*10^3	53*10^3	212*10^3
MPV (fl)	_	7	6.9	0	6.4

**Fig. 1:**
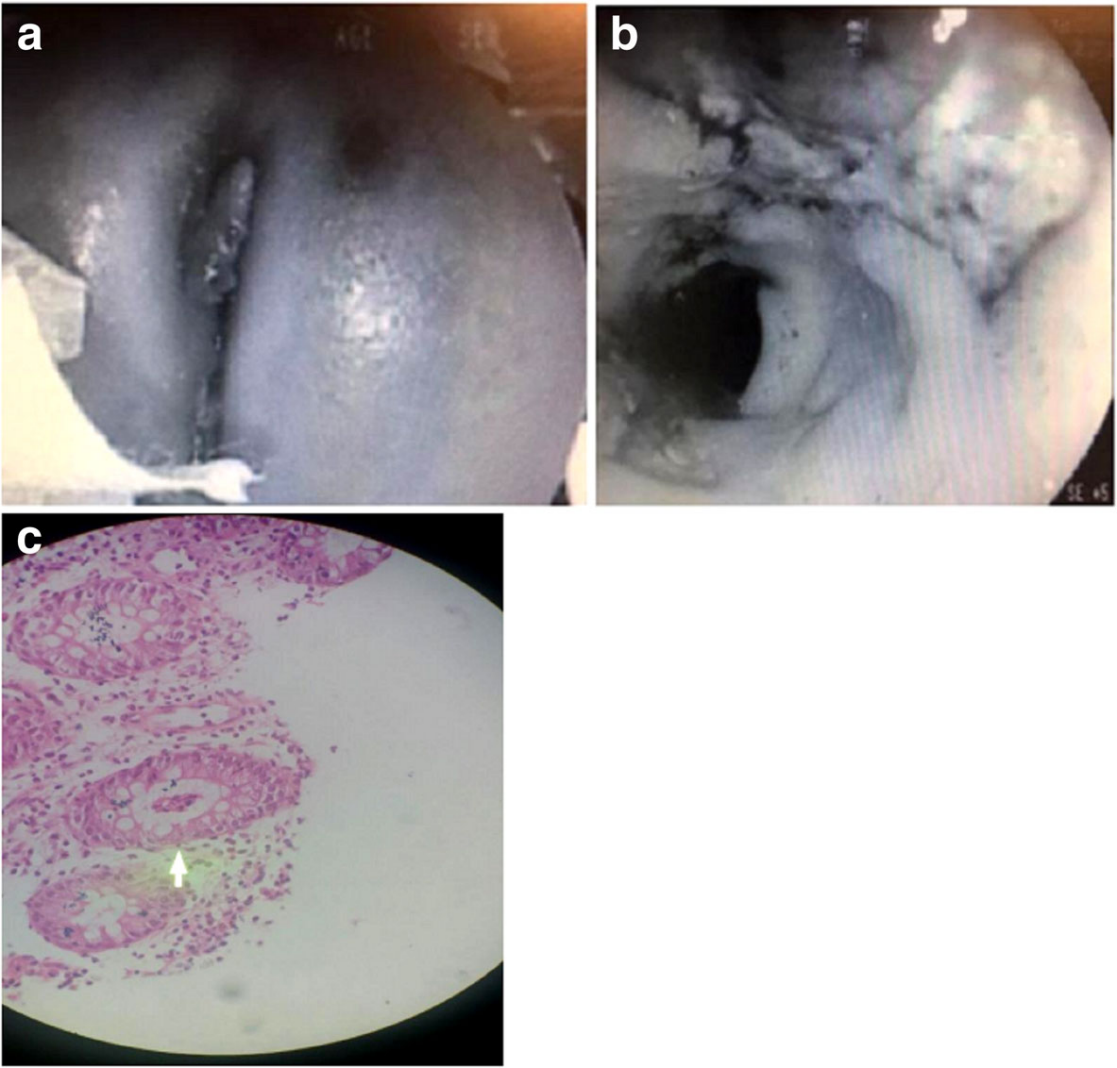
Sigmoidoscopy study in the proband. **a**. It is shown severe and diffused ulcer, polypoid lesion and decreased vascularity in rectum. **b.** Colon and rectum biopsy revealed chronic colitis. **c**. The histological section from colon and rectum showed cryptitis and crypt abscesses (Arrow)

Based on the patient history and clinical and laboratory findings described above, WAS disease was clinically suspected; therefore, we performed immunological assays. For instance, flow-cytometry showed normal results but the level of antibodies for IgG, IgA, and IgE was high (IgM was in normal range) (Table 2). At that time, to control the sepsis, broad spectrum of antibiotics (Vancomycin and Meropenem) were administered for the patient. At the age of 8 months, the patient had poor feeding, abdominal distension, and lower gastrointestinal bleeding. At that time, the patient was febrile and he was in respiratory distress. Generalized skin petechia and perianal skin tag were also detected. Due to rectal bleeding, endoscopy and sigmoidoscopy were also performed and results revealed severe erythema, erosion, and nodularity in antrum of stomach and nodularity and erythema in the bulb of esophagus. In sigmoidoscopy, skin tag, fistula, fissure in the perianal area, severe and diffused ulcer, and polypoid lesion and decreased vascularity in rectum were detected (Fig. 1a). Colon and rectum biopsy showed chronic colitis with severe activity in favor of inflammatory bowel disease (Fig. 1b). Histological examination of biopsies from the patient also revealed cryptitis and crypt abscesses (Fig. 1c). Since there was no an infectious etiology, these observations were in support of chronic colitis and/or IBD. To find the genetic cause of the disease in our patient, we performed Next Generation Sequencing (NGS) technique to sequence all exons of protein-coding genes.

**Table 2:** Results of flow cytometry and immunoglobulin study in the proband at the age of 4 months

Test	Result	Unit	Reference
CD3	82.96	%	30–78
CD4	33.78	%	22–58
CD8	8.9	%	10–37
CD4/CD8	3.8	%	1–4
CD16	9.59	%	5–19
CD19	47.84	%	9–38
CD20	45.12	%	3–15
CD56	9.15	%	3–15
Dihydrorhodamine (DH)	90 (Normal Range > 50)		
IgG	11.73	g/L	1.8–8.0
IgM	0.615	g/L	0.20–1.0
IgA	1.25	g/L	0.08–0.8
IgE	713	U/L	> 10.0-Atopy possible > 50.0-Atopy high risk
Tetanus Ab IgG	1.43	IU/ml	< 0.1 Basic immunisation recommended 0.1–1 To be controlled after 1–2 years 1–5 To be controlled after 2–4 years > 5.0 To be controlled after 4–8 years

Whole Exome Sequencing (WES) using Illumina NextSeq500 for pair-end 150-nucleotide sequencing was performed on DNA sample from proband. The NGS results were analyzed using bioinformatics tools, including BWA aligner [12], GATK [13], Annovar [14], and open access bioinformatics tools. Totally, more than 120 K annotated variants were identified with hetero/homo ratio of 1.6 to 1.8, which were then filtered based on their frequency, position, functional consequences, pattern of inheritance and mainly clinical phenotype. NGS data of the proband, revealed a novel, private, hemizygous splice site mutation (NM_000377:exon3:c.360 + 1G > C, Chromosome X**)** in *WAS* gene. This identified mutation was not reported previously, therefore, is categorized as the variant of unknown significance (VUS). However, due to lack of any other mutation that can explain the phenotype in this patient, almost complete phenotypic correlation between the disease and identified mutation, and disrupting nature of splice-site mutations, we believe this is a pathogenic mutation. We will deposit into the Clinar database (submission number: SUB3969419). Additionally, as the mutation has no reported frequency in our database (Bayangene) or any other available public variant databases, it was considered as a private mutation.

**Fig. 2:**
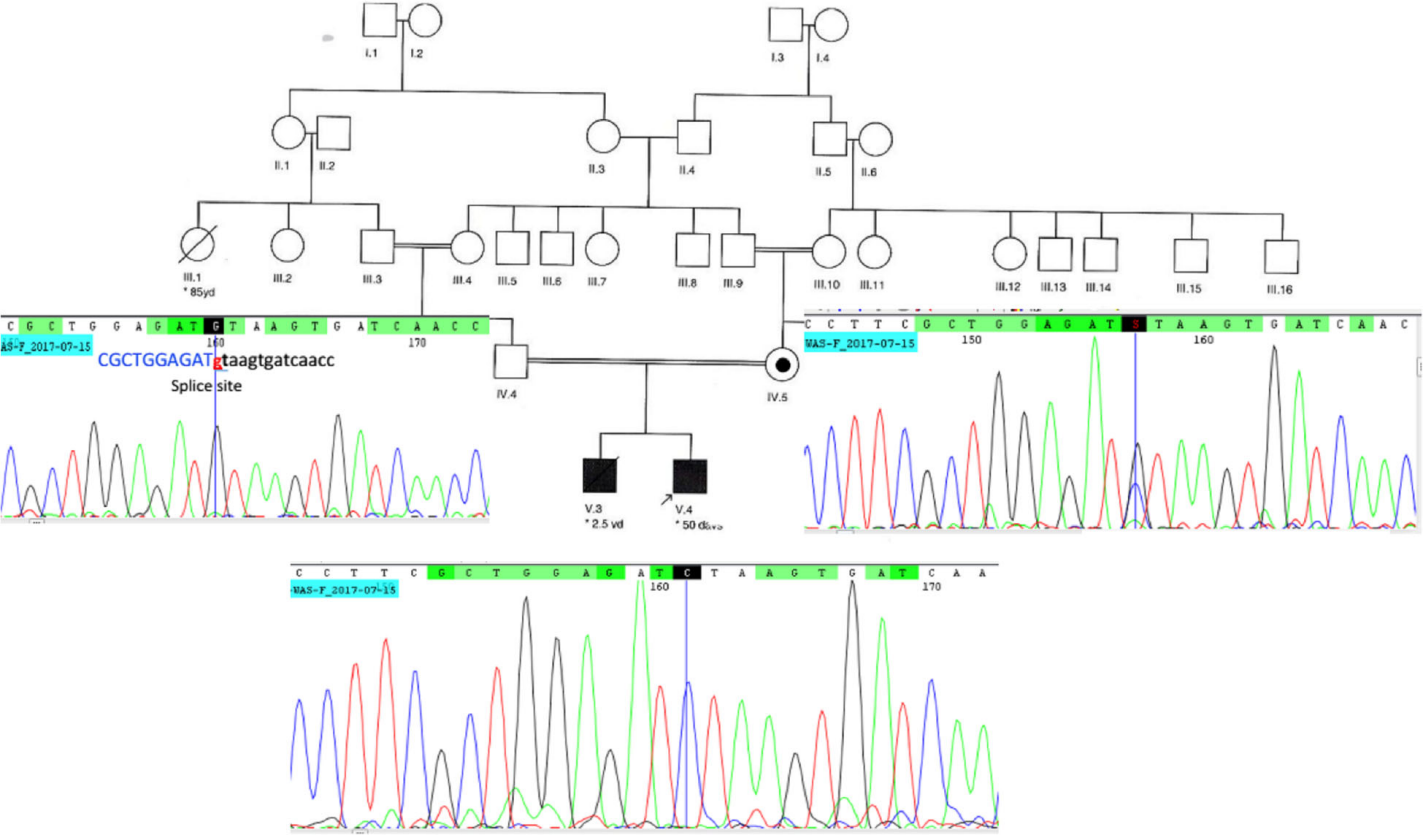
Sanger sequencing chromatogram. The proband showed hemizygous c.360 + 1G > C (C/C), his mother as heterozygous (G/C), and his father as normal (G/G)

To confirm the identified novel mutation, genomic DNA was prepared from whole blood samples of family members of the proband by QIAamp DNA Blood Mini Kit (Qiagen, Germany) according to the company’s protocol. Then, PCR was carried out for the proband and his parents using following primers: FWAS-E3: GCTCCCAAATCCAGACAC and RWAS-E3: CTTGCACTAGAGGACTCAC (PCR product: 561 bp) for amplification of exon 3 of *WAS* gene. After that, Sanger sequencing was performed for PCR products on 3130XL Genetic Analyzer (Applied Biosystems USA) according to ABI BigDye Terminator Cycle Sequencing Kit (Applied Biosystems, USA). Sanger sequencing results were analyzed with the use of NCBI BLAST and CodonCode Aligner software in which it confirmed hemizygous status in proband, heterozygous in his mother and normal in his father (Fig. 2). Different bioinformatics software and websites were also used to identify the features and the consequences of mutation in the given position of the protein and also to provide the family pedigree, including Human Splicing Finder (HSF, http://www.umd.be/HSF3/technicaltips.html), STRING (functional protein association networks, https://string-db.org), and Pedigree Chart Designer from CeGat (https://www.cegat.de). Following evidences can confirm that this mutation is led to WAS: 1- WES detected only this mutation to be linked with observed clinical and laboratory findings suggestive of WAS in the proband. 2- Sanger data given in Fig. 2, confirmed the presence of the mutation in the proband as hemizygous, his mother as heterozygous, and his father as normal, confirming the X-linked segregation of the disease. 3- Mutation is located within the donor splice site which is expected to be highly damaging since another mutation in this nucleotide (c.360 + 1G > T, with ID number of CS972885 and CS961711in HGMD) could affect splice site. 4- Using HSF tool, it predicted that the wild type (WT) splice site will be broken and alteration of the WT donor site, most probably affects splicing. 5- Exon 3 is within WH1 domain which is important for binding to a Pro-rich ligand; therefore, skipping of this exon can be very damaging and most probably cause the sever form of the disease [1].

In our study we also used STRING tool to predict the association network between WAS protein and other proteins. Its results revealed that WASp has interaction with several main proteins involved in different biological pathways and cellular component.

## Discussion and conclusion

WASp is a member of WAS family of proteins with similar domain named proline-rich region (PPP) which can bind by SH3 domains. WASp has also other essential domains and with identification of various motifs and regions across WASp, it has revealed that they can interact with key proteins in biological pathways. The domains are regulated by different factors such as GTPase, Cdc42 (role in the actin filament formation), ARP2/3 (the cytoskeletal organizing complex), p47nck, a 47-kD cytosolic adapter protein, and tyrosine-protein kinase Fyn [15, 16]. WASp is considered as a link between CDC42 and the actin cytoskeleton since in males affected with WAS, T- lymphocytes show a sever disruption of actin cytoskeleton, which may be due to the impaired Cdc42 signaling [16, 17]. Therefore, dysregulation of pathways linked to WASp have dramatic effects on different biological processes such as actin polymerization [16, 18, 19]. As described in result section, STRING tool showed that WASp has interaction with several key proteins, for example ARP2 actin-related protein 2 homolog, yeast, (ACTR2), WAS/WASL interacting protein family, member 1 (WIPF1), ARP3 actin-related protein 3 homolog, yeast, (ACTR3), actin related protein 2/3 complex, subunit 2, (ARPC2), cell division cycle 42 (GTP binding protein, 25 kDa) (CDC42), actin related protein 2/3 complex, subunit 3 (ARPC3) and so on. These interactions and associations with different proteins suggest the possible indirect involvement of WASp in various pathways and any interrupted interactions may cause life-threating clinical phenotypes. Therefore, the study of different mutations in this gene and functional effects of the mutations help understand the exact mechanism of this gene in different pathways, explaining why different mutations in this gene lead to some specific characterizations of the disease (for example the identified mutation in our study resulted in both WAS and chronic colitis with severe activity in favor of inflammatory bowel disease), and shed light onto the therapeutic approaches for this disease through its corresponding pathways.

In summary, a novel donor splice site mutation was found in *WAS* gene in our patient who had both WAS and chronic colitis suggestive of inflammatory bowel disease. Such researches may help consider the genetic counselling more precisely for families with clinical phenotypes suggestive of these diseases and help to uncover the biological pathways linked to *WAS* gene.

## Abbreviations

ACTR2: ARP2 actin-related protein 2 homolog, yeast,
ACTR3: ARP3 actin-related protein 3 homolog, yeast
ARPC2: Actin related protein 2/3 complex, subunit 2
ARPC3: actin related protein 2/3 complex, subunit 3
CDC42: cell division cycle 42
CRP: C-Reactive Protein
ESE: Exonic Splicing Enhancers
ESR: Erythrocyte Sedimentation Rate
Hb: Hemoglobin
HGMD: Human Genome Mutation Database
HSF: Human Splicing Finder
IBD: inflammatory bowel disease
ITP: idiopathic thrombocytopenic purpura
IVIG: intravenous immunoglobulin
MPV: mean platelet volume
NGS: Next Generation Sequencing
PPP: proline-rich region
VUS: variants of unknown significance
WAS: Wiskott-Aldrich syndrome
WASp: Wiskott-Aldrich syndrome protein
WES: Whole Exome Sequencing
WH1: Wasp homology 1
WIPF1: WAS/WASL interacting protein family, member 1
WT: Wild Type
XLN: X-linked neutropenia
XLT: X-linked thrombocytopenia
